# A Modified Surgical Technique to Prevent Parastomal Hernia

**DOI:** 10.3389/fsurg.2022.907316

**Published:** 2022-06-28

**Authors:** Zhuolin Fan, Weiyu Guan, Tao Zhang, Dewei Zhang

**Affiliations:** Department of General Surgery, The Fourth Affiliated Hospital of China Medical University, Shenyang, China

**Keywords:** colostomy, parastomal hernia, posterior rectus abdominis sheath, abdominal rectus muscles, rectal cancer, abdominoperineal resection

## Abstract

An extraperitoneal colostomy is not sufficiently effective in preventing parastomal hernias. On the basis of anatomic structures and mechanical principles, we modified this surgical technique by preserving the integrity of the posterior rectus abdominis sheath to prevent parastomal hernia, and we applied it clinically.

## Introduction

Parastomal hernia (PH) is inevitable after a colostomy. Because of the defect in the abdominal wall at the stoma site, the incidence rate is estimated to be more than 30% at 12 months, 40% at 2 years, and 50% or higher for a longer follow-up period (1, 2). Repair of the resulting obstruction or strangulation is difficult (3). Therefore, it is critical to improving surgical techniques to prevent PH.

There is currently no consensus about the surgical technique to prevent PH. The use of mesh is recommended by the European Hernia Society (2), but some researchers showed that a larger clinical trial is required to support its use (4). Some side effects, including infection, intestinal adhesion, and intestinal perforation, as well as the procedure’s high cost and uncertain effectiveness, have caused the indications for mesh use to become stricter (5). Therefore, improvement in surgical techniques is required to prevent PH. The extraperitoneal colostomy technique was first proposed in 1958 (6), and it is widely used clinically (6–9). The PH incidence rate using this surgical technique was reported to be lower than that of other techniques (10–12). However, taking into consideration the natural anatomy of the abdominal wall, this technique requires further improvement (13). Therefore, on the basis of the Sugarbaker PH repair and incisional hernia surgery (14) and considering the importance of the posterior rectus sheath (15, 16), we modified the extraperitoneal colostomy surgical technique. In this paper, we introduce the surgical procedures and the principles in detail.

## Surgical Procedures

### Step 1. Preparation for Surgery

**Figure 1:**
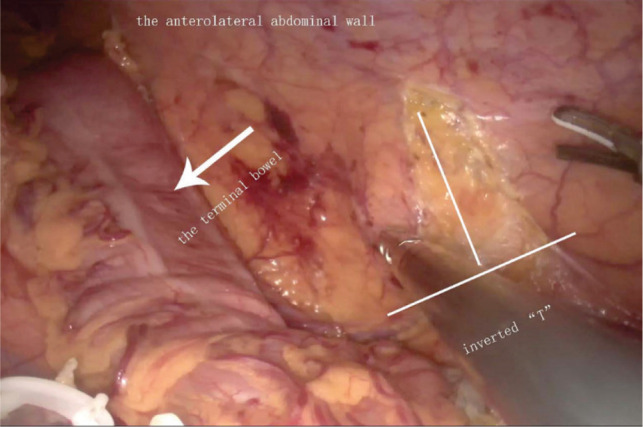
Tunnel entrance.

Before surgery, the stoma site was marked 2.0–3.0 cm to the left of the umbilicus to ensure that the stoma would be covered by the abdominal rectus muscles (Figure 1) and to facilitate postoperative nursing care. The preparatory procedures involved in step 1 are the same as those followed for abdominoperineal resection.

### Step 2. Tunnel Establishment

**Figure 2:**
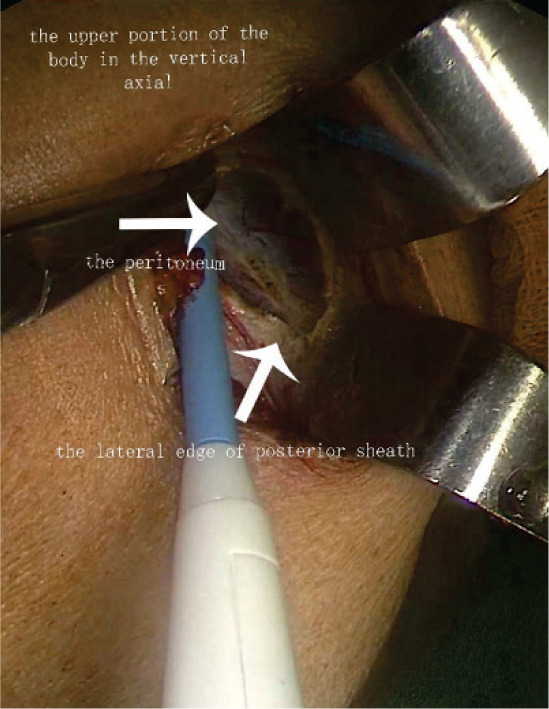
Open approach.

**Figure 3:**
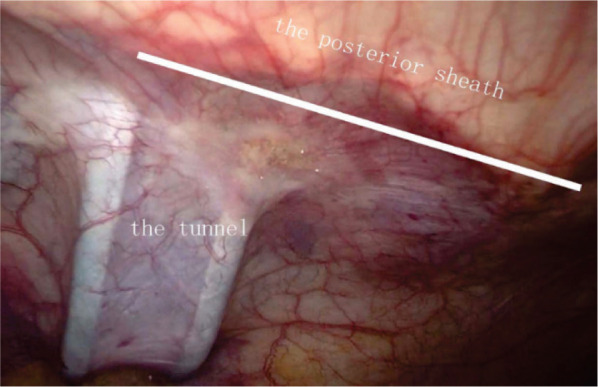
Extraperitoneal tunnel.

To establish the tunnel, the left anterolateral abdominal wall is located by marking the skin and using laparoscopic monitoring. The transversalis fascia and peritoneum are then opened in an inverted T shape, which is the tunnel’s entrance. As indicated in Figure 1, the horizontal line of the “T” is parallel to the anterior axillary line, while the vertical line indicates the stoma tunnel that extends to the anterior axillary line. Pneumoperitoneum pressure is eliminated, and a circular skin disc that is approximately 2.5 cm in diameter is fashioned on the basis of the marked skin site. The diameter of the circular skin disc can be adjusted, but it should not exceed 3.0 cm (Figure 2). The anterior rectus sheath is opened in a cross shape. Then, the abdominal rectus muscles are split down the center to expose a sufficient amount of the posterior sheath, at which point the lateral edge of the posterior sheath can be visualized. A vertical incision that is approximately 6.0 cm in length is created in the posterior sheath approximately 0.5 cm adjacent to the lateral edge (Figure 2). Under different bowel conditions, caution is needed to maintain the blood supply and establish a smooth path through which the bowel can pass. Then, the low pneumoperitoneum is re-established, and the tunnel beneath the posterior sheath is established using ring forceps and blunt dissection. The orientation aligns with the vertical line that was previously mentioned. Using this approach, the integrity of both the posterior sheath and the peritoneum can be maintained. The total length of the tunnel should be approximately 8.0–10.0 cm (Figure 3).

### Step 3. Stoma Creation

**Figure 4:**
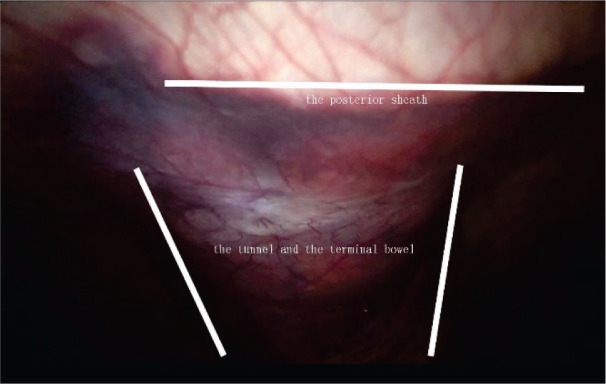
Stoma route.

**Figure 5:**
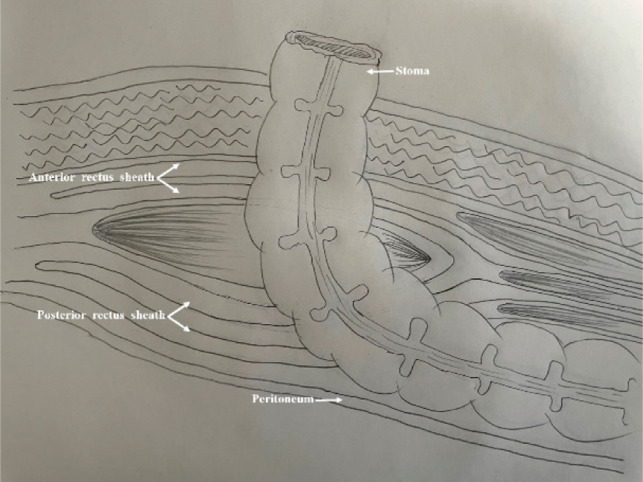
Anatomical sketch (left side).

The bowel is exposed and pulled out of the abdominal cavity through the tunnel and the incision in the posterior sheath using the ring forceps. Care should be taken to avoid twisting the intestines and mesenteries, and the posterior sheath integrity should be checked to ensure that there is no bowel entrapment (Figures 4, 5 and Supplementary Video). When necessary, a few stitches can be placed for reinforcement on both sides of the tunnel entrance using a 3-0 polydioxanone suture, and the stoma is created using triplicate sutures. The first suture is placed through the peritoneum, the anterior and posterior rectus sheath, and the seromuscular layer of the cut end of the bowel, followed by a seromuscular bite, and then through the dermal layer. The stoma should then be opened, and full-thickness bites of the end of the bowel and the dermis of the skin are performed at 0.3 cm intervals along the edge of the stoma.

## Principle Discussion

### Anatomic Factors

The rectus sheath, which is the major functional structure in the abdominal wall, is a durable, resilient, and fibrous compartment (16). It is critical to properly close the surgical incision. Therefore, the rectus sheath composition is of great significance for surgeons (15–17). Maintaining the integrity of the rectus sheath is important, both in theory and in practice. In a previously described technique, the posterior rectus sheath is incised vertically and transversely (6). In our technique, however, the posterior rectus sheath under the stoma is kept as intact as possible. Moreover, the strength of the abdominal wall under the stoma is reinforced. Because the posterior rectus sheath below the arcuate line is weak, we opened the stoma approximately 2–3 cm to the left of the umbilicus, ensuring that the stoma would be located above the arcuate line (16). We then made an incision along the lateral edge of the posterior sheath to maintain the integrity of the posterior sheath, which is the major functional and important structure of the abdominal wall (15, 18). Moreover, the integrity of the lateral edge of the anterior sheath was also maintained to preserve the blood and nerve supply to the abdominal rectus muscles and maintain the original function of the abdominal rectus muscles (17). Next, we placed the stoma through the abdominal rectus muscles to strengthen the stoma and prevent retraction and prolapse. Preservation of the blood and nerve supply using this technique reduces the possibility of abdominal rectus muscle atrophy.

### Mechanical Principles

A complete abdominal wall is essential for patients to maintain stable intra-abdominal pressure and counteract pressure changes (13). Using conventional techniques, the bowel is at approximately a right angle to the rectus sheath. An extraperitoneal end tunnel colostomy before the abdominal rectus muscle posterior sheath was recently proposed by surgeons to maintain an obtuse angle, which facilitates the stoma–bowel attachment to the tunnel wall and alignment with the direction of mechanical conduction; this reduces retraction and longitudinal tensile force (14). Because the extraperitoneal end tunnel colostomy has several advantages, we modified this method. We took the integrity of the posterior sheath into account. The intra-abdominal pressure that is exerted onto the stoma site will be mitigated on the basis of the integrity of the posterior sheath. Additionally, the strength of the inner abdominal wall is more strongly reinforced when using the proposed technique than when using the standard extraperitoneal end tunnel colostomy technique, which does not maintain the integrity of the posterior sheath. This dual advantage means that the incidence of PH is significantly reduced (12). The abdominal rectus muscles surrounding the stoma will also function as a sphincter, and the integrity of the lateral edge of anterior sheath is retained to preserve the partial blood and nerve supply to the abdominal rectus muscles (18).

### Conclusion

PH is the most common complication of colostomy, and its incidence increases with age (19, 20). Serious outcomes place mental and physical burdens on patients. Therefore, establishment of a surgical technique to prevent PH is critical. Stoma creation using a mesh to prevent PH seems to be a reasonable solution; however, it is expensive, and its uncertain prognosis hinders its development (2, 4). Because there is little evidence for the effectiveness of extraperitoneal colostomy in preventing PH (2), we modified this surgical technique by performing an extraperitoneal end tunnel colostomy at the lateral edge of the posterior sheath. An intact posterior sheath increases the intra-abdominal pressure that is exerted on the posterior rectus sheath and reduces the axial force of the bowel stoma, which is similar to the principle of the Sugarbaker PH repair technique (14, 21). Additionally, the posterior sheath, which is an important structure within the abdominal wall (12, 17), is used as another layer under the stoma to strengthen the abdominal wall. Thus, we preserved the integrity of the posterior sheath to reinforce the defect in the abdominal wall at the stoma site.

At our institution, increasing numbers of patients are undergoing surgery using this modified surgical technique. We confirmed that the incidence of PH decreased during the short-term follow-up (12). Because we identified certain persistent limitations, we further improved this technique and standardized the procedures and details. Therefore, this modified surgical technique is a potential strategy to prevent PH after a colostomy. However, long-term follow-up of patients who have undergone this procedure is required.

## Data Availability

The raw data supporting the conclusions of this article/Supplementary Material will be made available by the authors, without undue reservation.
